# Microbiome resilience of three-toed box turtles (*Terrapene carolina triunguis*) in response to rising temperatures

**DOI:** 10.3389/fvets.2024.1276436

**Published:** 2024-09-02

**Authors:** Jimmy Guan, Gustavo A. Ramírez, Curtis Eng, Brian Oakley

**Affiliations:** ^1^College of Veterinary Medicine, Western University of Health Sciences, Pomona, CA, United States; ^2^Department of Biological Sciences, California State University Los Angeles, Los Angeles, CA, United States

**Keywords:** microbiome, chelonians, zoonosis, global warming, *Erysipelothrix* spp.

## Abstract

The gastrointestinal (GI) microbiome of chelonians (testudines) plays an important role in their metabolism, nutrition, and overall health but the GI microbiome of three-toed box turtles (*Terrapene carolina triunguis*) has yet to be characterized. How the GI microbiome responds to rapidly rising environmental temperatures has also not been studied extensively in ectotherms, specifically chelonians. In this study, twenty (20) *T.c.triunguis* were split into control and experimental groups. The experimental group experienced 4.5°C increases every two weeks while the control group stayed at a constant ambient temperature (24°C) through the entirety of the experiment. Before each temperature increase, all turtles had cloacal swab samples taken. These samples underwent DNA extraction followed by 16S rRNA gene sequencing and microbial community analyses. Differences in diversity at the community level in the controls compared to the experimental groups were not statistically significant, indicating microbiome resilience to rapid temperature changes in *T.c.triunguis*, although some differentially abundant lineages were identified. Interestingly, an amplicon sequence variant belonging to the *Erysipelothrix* spp. was exclusively enriched in the highest temperature group relative to controls. Overall, our work suggests that there may be an innate robustness to rapid temperature swings in the microbiome of *T.c.triunguis* which are native to temperate North America. Despite this resilience, *Erysipelothrix* spp. was enriched at the highest temperature. Phylogenetic analysis of this amplicon variant showed that it is a close relative of *Erysipelothrix rhusiopathiae,* a pathogen of zoonotic importance associated with both wildlife and livestock.

## Introduction

The GI microbiome is a complex community of microorganisms that are a vital part of the hosts’ health, acting as a symbiosis contributing to various functions such as energy metabolism, immune system modulation, behavior, and even neurodevelopment (1, 2). These complex communities are directly tied to the health and disease of the host. Although numerous studies have been able to provide valuable insights into the GI microbiome in mammals (3–6), such studies have yet to be done extensively on reptiles, specifically members of the order *Testudines*, commonly referred to as turtles and tortoises. Throughout this study we will use the names chelonian and testudines interchangeably for the name of the taxonomic order containing all turtles and tortoises. *Testudines* currently contains 357 recognized species, over half (51%) of which are considered globally threatened. This makes turtles and tortoises one of the most threatened groups of vertebrates (7). Members of this order can be roughly divided into aquatic, semi-aquatic, and terrestrial species. Microbial community studies have been done on various aquatic species such as the green sea turtle (*Chelonia mydas*) (8), eastern-short neck turtle (*Emydura macquarii macquarii*) (9), red eared slider (*Trachemys scripta elegans*), and Chinese three-keeled pond turtle (*Chinemys reevesii*) (10) as well as terrestrial species such as Aldabra giant tortoises (*Aldabrachelys gigantea*), gopher tortoises (*Gopherus polyphemus*), and Bolson tortoises (*Gopherus flavomarginatus*) (11). Interestingly, the microbiome of semi-aquatic turtles has yet to be characterized.

Three-toed box turtles (*Terrapene carolina triunguis*) are semi-aquatic turtles belonging to the genus *Terrapene.* These terrestrial turtles are found throughout North America, characterized by a movable hinged plastron that can completely close (12). *T.c.triunguis* spends most of the time on land among ground covers that aid in thermoregulation, and generally only enter bodies of water during periods of high temperatures and drought (13). Like all ectotherms, *T.c.triunguis* relies entirely on environmental temperatures in order to thermoregulate. Because ambient temperature directly affects body temperature, environmental temperatures have a strong influence on ectotherm physiology, behavior, and performance (14). The rapidly warming climate of the Anthropocene (15) challenges *T.c.triunguis* and other turtles/tortoises with these unnatural thermal increases. Climate change has had important documented effects on the individual, population, and community ecology of *Testudines* (16), but the effects of increased ambient temperature on the GI microbiome, to our knowledge, have not been previously examined.

As an ectotherm, *T.c.triunguis* and its microbiota are particularly susceptible to fluctuations in temperature. Temperature-driven microbiome changes may alter the abundance of key lineages, including potential human and animal pathogens, affecting turtle health. Ambient temperature increases are known to perturb host-associated microbial communities in ectotherms and can result in dysbiosis and possible shedding of pathogens. For example, recent observations made in wild western fence lizards (*Sceloporus accidentalis*) exposed to increasing temperatures showed significant changes in their gastrointestinal microbiome, including overall increases in putative pathogenic clades (17).

The objective of this study was to understand the effects of increasing ambient temperature on the gastrointestinal microbiota in *T.c.triunguis*. The changes observed provide important insight into microbial changes in a clade of vertebrates that are globally threatened and highly susceptible to heat stress. Not only is this important to animal health, but there are important public health implications. Both captive and wild-caught reptiles that are asymptomatic have been shown to harbor and excrete a wide array of pathogens that could impact human health. Common turtle-hosted enteric bacteria that are of zoonotic importance include *Salmonella* spp.*, Escherichia coli, Kiebsiella* spp., *Campylobacter* spp., and *Yersinia* spp. (18). As reptiles, especially turtles/tortoises, become more popular to keep as pets, this study may inform human and veterinary medicine from a One Health perspective about how rapidly changing environmental temperatures can affect both reptiles and humans.

## Methods

### Animal husbandry

Over twenty adult *T.c.triunguis* were obtained from a local turtle/tortoise rescue and housed together in a large group for 2 weeks before the start of the experiment in order to ensure they were equally exposed to the same environment and diet and were considered healthy for the study. All animals were medically evaluated by an experienced exotic animal veterinarian (CE), and any turtles that were deemed unhealthy were removed prior to the start of the study. Blood samples could not be taken due to funding limitations and lack of manpower as this study occurred during the SARS-CoV2 pandemic. Twenty turtles were split equally between males and females into control and experimental groups. Sex was presumed based on sexual dimorphism; females had brown iris color, shorter tails and a flat plastron shape while males had red to orange iris color, longer tails and had a more concave shaped plastron (19). One turtle experienced lethargy, inappetence, and blepharitis during the first week of the study and was removed. The removal of one turtle during the study resulted in the control group with 9 turtles and the experimental with 10. The two groups were housed in separate 50-gallon Rubbermaid stock tanks covered with heavy-duty plastic to create and maintain a microenvironment providing consistent lighting, temperature and humidity. Turtles were housed on 5″ deep cypress mulch substrate (ZooMed Forest Floor) and allowed free access to water. All turtles were free fed as a group approximately 200 g of pellets (Mazuri® Tortoise Diet) supplemented with approximately 100 g of thoroughly rinsed mixed greens, 10–15 red wriggler worms, and powdered calcium (ZooMed ReptiCalcium) three times a week. All diets fed to both groups were obtained from identical sources. UVB lighting was provided 12 h a day using four-foot-long T-5 high output UVB light fixtures (ZooMed ReptiSun).

Five 100-watt ceramic heat emitters (ZooMed Repticare Ceramic Infrared Heat Emitter) were placed approximately 50 cm above the enclosures and connected to digital thermostat controllers (ZooMed ReptiTemp). The control group was maintained at 24°C for the duration of the experiment. This temperature was considered within the lower range of the preferred optimal temperature zone (POTZ) for this species (20). The experimental group experienced 4.5°C increases in temperature every two weeks from 24°C to 28.5°C and finally 33°C. The final temperature point was considered within the higher range of the POTZ (20) without the possibility of resulting in hyperthermia since animals would be exposed to this temperature consistently. Whereas, in the wild animals have the ability to seek shade or swim into deeper areas where temperatures are noticeably cooler. Humidity was maintained between 50–60% in both enclosures for the duration of the experiment. Ambient temperature and humidity were monitored by three different digital temperature and humidity gauges (ZooMed) and recorded daily. Carapace and plastron temperatures were also taken on each turtle with an infrared thermometer (ZooMed ReptiTemp) and recorded daily. The carapace and plastron temperatures were averaged to obtain accurate total body temperature. Appetite, activity, health, and hydration were subjectively recorded three times a week on a plus-minus scale. All animals were given free access to any location in the enclosure to allow for behavioral thermoregulation. Any turtles that spent a subjectively large amount of time under a heat source or burrowed in the substrate were noted and confirmed to have higher or lower than expected body temperature, respectively. Weights were recorded twice a week with a precision scale.

### Sample collection and DNA purification

Every two weeks, before increasing the ambient temperature in the experimental group, all turtles had cloacal swab samples taken. The perineal area was wiped down with square gauze and 70% isopropyl alcohol to clean and disinfect the site. Thermo Scientific™ Remel BactiSwab™ culturette swabs were inserted into the cloaca until the cotton portion was not visible and spun 4–5 times for adequate sample collection. The swabs were placed into the provided culturette tubes with a sponge soaked in liquid transport medium and stored at −20°C. Within 3 months of collection, the stored samples were slowly thawed in an ice bath for DNA extraction. The entire cotton tip and liquid transport medium were used to extract and purify DNA with the QIAmp® PowerFecal® Pro DNA kit. One uL of purified DNA was placed onto a Thermo Scientific NanoDrop™ for nucleic acid quantification. The purified DNA samples were stored at −20°C.

### 16S rRNA amplicon sequencing, amplicon sequence variant (ASV) generation, and statistical analyses

16S rRNA genes were targeted from DNA samples via PCR using the 519F and 926R (V4-V5 region) primers as previously described (21). Raw sequence Illumina fastq files are deposited in the NCBI SRA archive under the following BioProject number: PRJNA924021.

Amplicon Sequence Variants (ASVs) were generated from high-quality 16S rRNA gene sequences using Divisive Amplicon Denoising Algorithm 2 (DADA2) (22) implemented in the RStudio software. Briefly, forward and reverse reads were trimmed with the filterAndTrim() command using the following parameters: trimLeft = c(20,20), maxEE = c(2,2), rm.phix = TRUE, multithread = TRUE, minLen = 130, truncLen = c(290,200). Subsequently we performed an error assessment followed by independent dereplication of forward and reverse reads. Sequence error removal was performed with the dada() command. Next, error-free forward and reverse reads were merged using the mergePairs() command. Prokaryotic 16S rRNA gene Amplicon Sequence Variants (ASVs) were assigned taxonomy using the SILVA 132 database. ASV-based community composition was visualized in R using ggplot2 implemented in Phyloseq (23) and custom plot scripts.

Microbial community differences between (experimental and control groups) and within (differences among week sampling points) treatment groups were explored with permutational multivariate analysis of variance using the ADONIS function of the R package Vegan (24) implemented on Bray-Curtis distances. Final probabilities were adjusted for multiple comparison inflation by implementing Benjamini-Hochberg correction via the p.adjust (24) function in R. Differential abundance analysis of microbial taxa was performed using the R package *DeSeq2* (25). Specifically, per ASV count data was fitted to a negative binomial distribution model which allows coefficients (log2-fold counts) and standard error estimates for each sample group. A Wald Test, using maximum likelihood estimates of our ASV model coefficients, was then used to test (Pval = 0.05) the following hypothesis between sample groups: *H_0_* = *no differential abundance*.

### Phylogenetic analysis

A comparative phylogenetic analysis of the Eysipelotrichaceae family 16S rRNA gene was performed using the sequence alignment program Multiple Alignment Using Fast Fourier Transform (MAFFT) (26) with the following command parameters: mafft --maxiterate 1,000 –localpair seqs.fasta > aligned.seqs.fasta. Maximum likelihood trees with 100 bootstrap supports were constructed with the Randomized Axelerated Maximum Likelihood (RAxML) program (27) as follows: raxmlHPC -f a -m GTRGAMMA -p 12345 -x 12,345 -# 100 -s aligned.seqs.fasta -n T.tree, −T 4 ML search + bootstrapping. Newick tree files were uploaded to FigTree v1.4.2 for visualization. An *E. coli* (accession: AB681728) sequence was used as the tree outgroup.

## Results

### Control vs. experimental measurements

**Figure 1:**
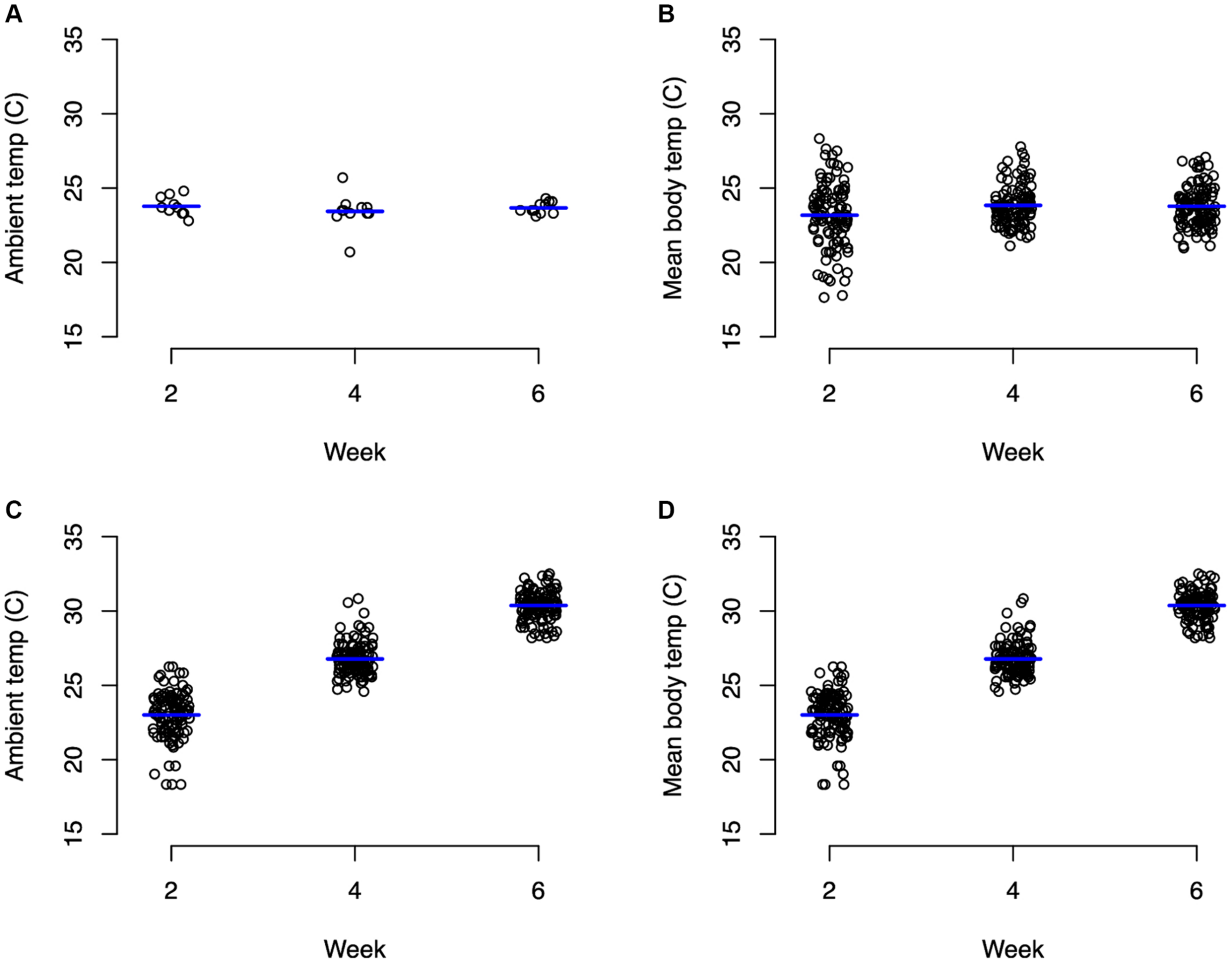
Strip charts of recorded ambient and body temperatures in Celsius. **(A)** Represents ambient temperature for animals in the control group, **(B)** represents body temperature for control turtles, **(C)** represents ambient temperature for animals in the experimental group, and **(D)** represents body temperature for experimental turtles. Significant (*p* < 0.0001) differences in ambient and body temperature for the experimental group between every time point.

**Figure 2:**
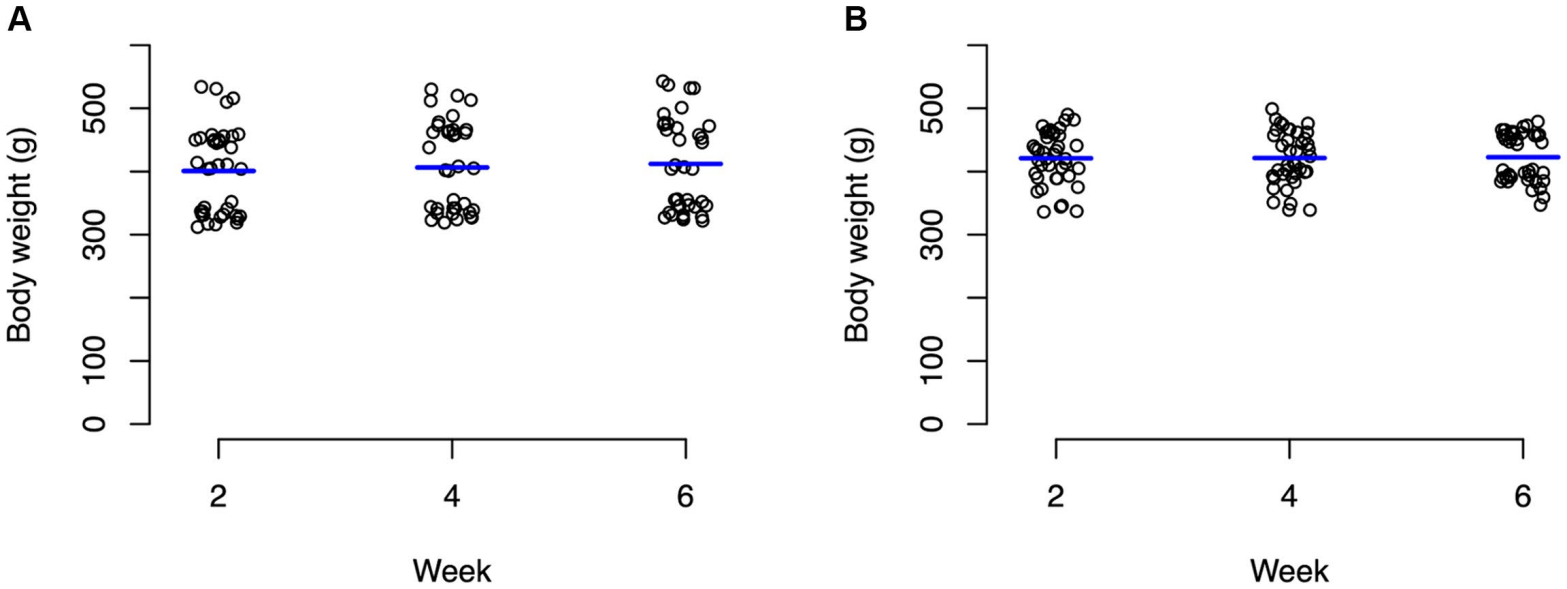
Strip charts of baseline body weights of all turtles in grams. **(A)** Control turtles and **(B)** experimental turtles. No significant changes in weight were observed in both groups through the entirety of the experiment.

For the control group, ambient temperature and body temperature did not change significantly through the course of the experiment, with an average ambient temperature of 23.6°C (Figure 1A) and average body temperature of 24.3°C (Figure 1B). In contrast, the experimental group experienced significant changes (*p* < 0.0001) in both ambient temperature (Figure 1C) and body temperature (Figure 1D). For weeks 1–2, body temperature averaged 22.9°C and ambient temperature averaged 24.8°C. During weeks 3–4, body temperature averaged 26.7°C and ambient temperature averaged 28.5°C. During weeks 5–6, body temperatures averaged 30.4°C and ambient temperature averaged 33°C (Figure 1). Average body weights at the beginning of the experiment were not significantly different between the control animals (mean 400.8 g) and the experimental animals (mean 420.9 g) with neither group changing significantly throughout the experiment (Figure 2; Supplementary Table S1).

### Community analysis

**Figure 3:**
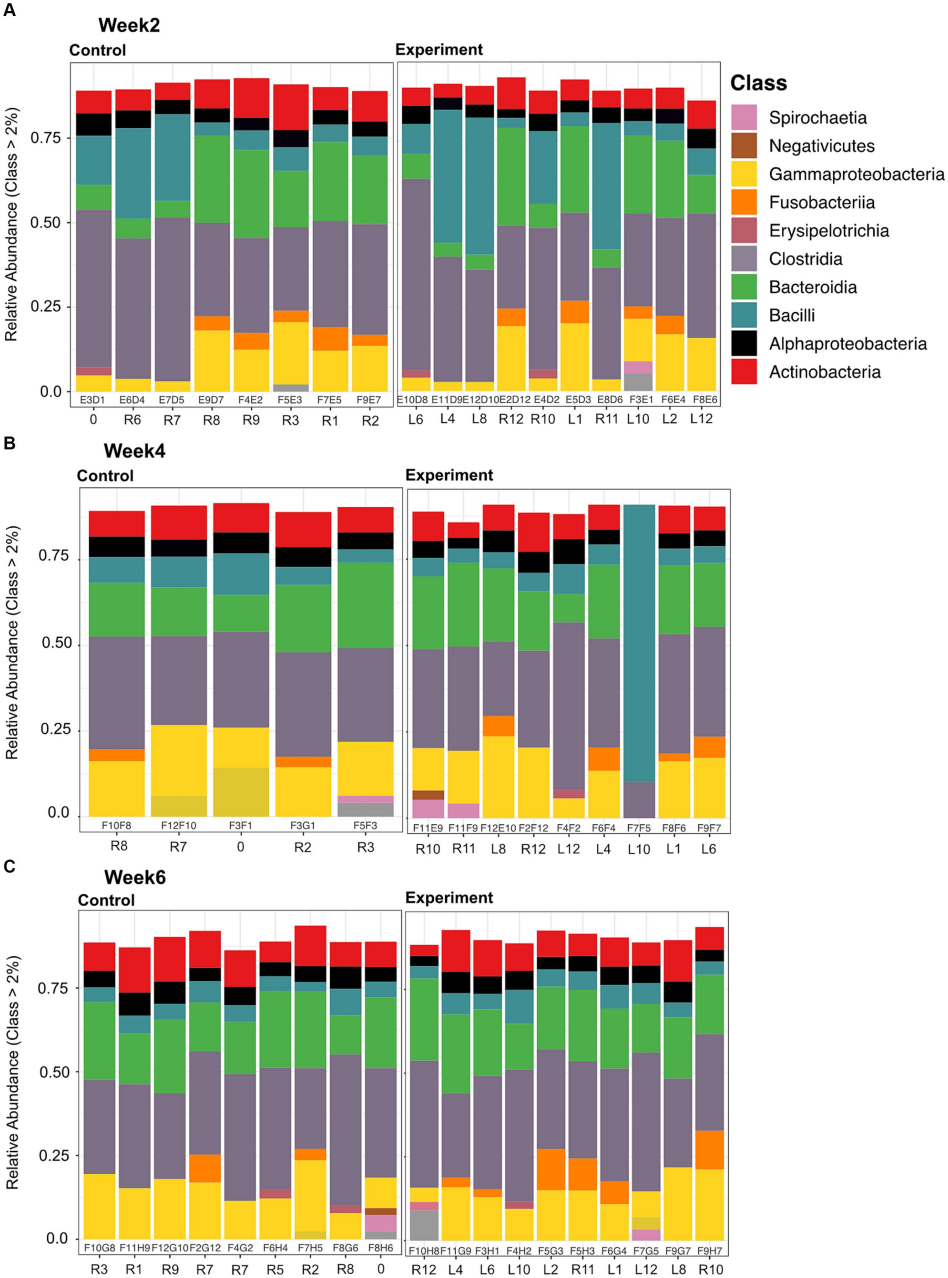
Stacked bar plots of relative abundance at the class level for samples collected between control and experimental at each time point (Weeks 2, 4, and 6 depicted in panels **A**, **B**, and **C**, respectively). Each bar plot is labeled with a sequencing ID (within the box) and a Turtle ID (outside the box). Predominant classes present between all samples are: *Alphaproteobacteria*, *Gammaproteobacteria*, *Bacteroidia*, *Clostridia*, and *Bacilli*. Alpha numerical column labels within the black outline boxes represent sequencing identifier. For column labels outside the boxes, the “R” stands for right and “L” stands for left, denoting marking of the carapacial marginal scutes in order to identify individuals (e.g., R8 -> right scute 8), “0” is a turtle we marked on the middle scute.

Microbial community composition for both experiment and control groups across all time/temperature steps was predominantly (>75% relative abundance) comprised of *Firmicutes*, *Bacteroidetes*, and *Proteobacteria* phyla members (Supplementary Figure S1). More specifically, members of the following classes within these three phyla were dominant: *Alphaproteobacteria*, *Gammaproteobacteria*, *Bacteroidia*, *Clostridia*, and *Bacilli* (Figure 3).

**Figure 4:**
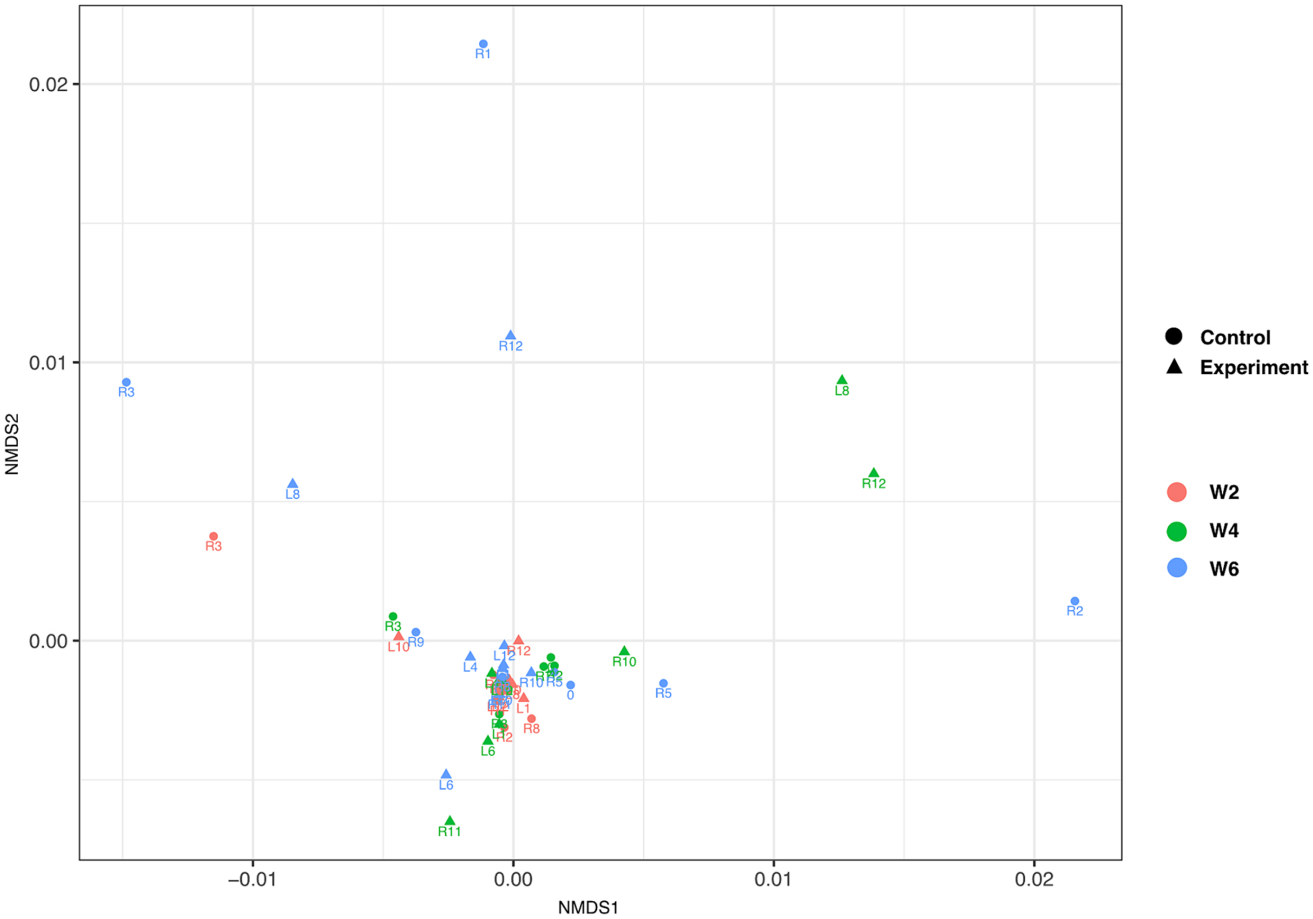
Non-metric multidimensional scaling of individual turtles. Triangles and circles depict experimental and control samples, respectively. Collection times week 2, week 4, and week 6 are depicted in red, green, and blue color, respectively. The data, based on Benjamini-Hochberg corrected permutational multivariate analysis of variance (PERMANOVA) *p* < 0.01, failed to show experiment group- or time of collection-driven structuring.

Alpha diversity metrics showed no significant differences (student *t*-test, Pval >0.05) between control and experimental groups at any collection time (Supplementary Figure S2). Similarly, non-metric multidimensional scaling (NMDS) of all turtles across all the time points showed no significant structuring [Benjamini-Hochberg corrected permutational multivariate analysis of variance (PERMANOVA) *p* < 0.01; Figure 4] between control and experimental groups at any collection time. No temperature driven changes were noted in stress-related organisms; however, there were high degrees of variation within both control and experimental groups at week 2 (Supplementary Figure S3).

### Enriched genera

**Figure 5:**
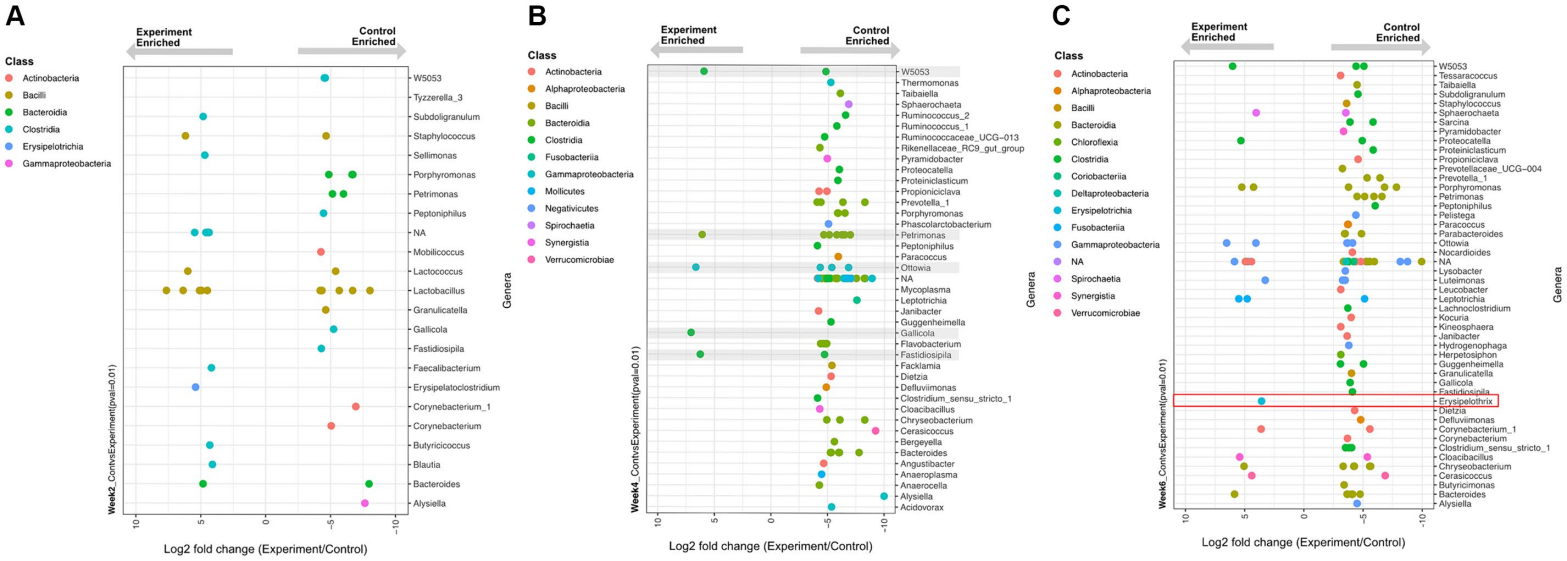
Differentially enriched (Wald Test, *p* < 0.01) genera between experimental and control group samples at time **(A)** week 2, **(B)** week 4, and **(C)** week 6. Each dot represents a differentially enriched ASV with experiment and control enrichment for negative and positive Log2 fold-change values, respectively. Each ASV is color coded as a function of taxonomic class and plotted on rows that represent genus-level assignments. **(B)** Grey boxes depict five ASVs enriched under experimental conditions. **(C)** The specific sequence type of *Erysipelothrix* spp. enriched in experimental group is highlighted in the red box.

**Figure 6:**
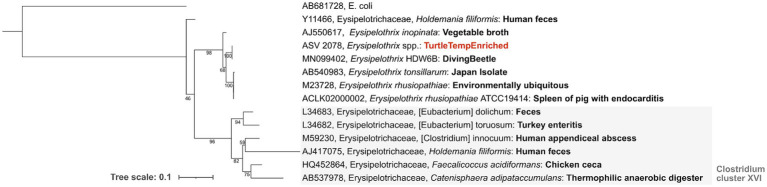
*Erysipelothrix* spp. phylogenetic tree (100 bootstraps iterations) for various environmental- and host-associated members of this genus. Sequence type ASV 2078, shown in bold red, is the *Erysipelothrix* spp. lineage differentially enriched in week 6 under experimental conditions relative to the control. An *E. coli* sequence (accession AB681728) was used as an outgroup.

Despite its non-significant community-level effect, temperature change elicited significant differential abundances (Wald Test, Pval <0.01) of some individual ASV lineages in the experiment versus control groups (Figure 5). This temperature-driven differential abundance trend between the control and experimental groups was strongest in week 4. Interestingly, despite most of the genera that became more abundant in the controls belonging to known commensal organisms, a temperature enriched ASV in week six was identified as a member of the *Erysipelothrix* spp., a well-known genus for pathogenic and zoonotic members. A genus-specific phylogenetic analysis of this ASV placed it in a sister clade to the *Erysipelothrix* Clostridium cluster XVI (Figure 6).

## Discussion

This study provides insights into the temperature-induced GI microbiome dynamics of semi-aquatic turtles, a group of largely understudied *Testudines.* All turtles in the experimental group experienced a significant change in ambient-induced body temperature at each time point. Three animals from the control group and two from the experimental group were removed from the data set because they experienced prolonged higher or lower temperatures than expected due to observed behavioral thermoregulation. Despite some individual lineages being identified as differentially abundant due to temperature change (Figure 5), the magnitude of temperature change and/or the total exposure time in our experiment failed to elicit significant diversity or community composition changes in the experimental relative to control groups (Figures 3, 4; Supplementary Figure S1). Our results contrast with other studies investigating effects of warming climates on the vertebrate and invertebrate gastrointestinal microbiome where significant decreases in both diversity and community structure have been reported (28, 29).

*T.c.triunguis* are commonly found in the midwestern and southern United States (e.g., Alabama, Arkansas, Georgia, Illinois, Kansas, Louisiana, Mississippi, Missouri, Oklahoma, Texas) (7) which experience a wide range of temperature differences from periods of extreme cold to extreme heat. Free ranging box-turtles have been observed to be active at temperature ranges from 11.0°C to 36.0°C (30). Unlike most reptiles, ectotherms native to these areas must be able to rapidly adapt to temperature swings. These adaptations may include rapid changes to their microbiome or alternatively, as reported here, a microbial community that exhibits minimal change despite environmental temperature shifts. The lack of significant community-level change between the two groups in our experiment could be due to the fact that the microbiome of *T.c.triunguis* has a level of innate robustness against rapid temperature swings.

Recent work with active-season ground squirrels showed that the microbiome did not change at different timepoints in the seasons, which the authors identified as a potential insensitivity to change of the obligate hibernator microbiome (31). Similarly, metatranscriptomic work on arctic ground squirrels shows that active season cecal microbiome composition is insensitive to seasonal time of collection and high versus low fat diet, however, both variables indeed affect microbiome transcriptional patterns (32). These hibernating mammal studies, despite not being directly comparable to our ectotherm study model, suggests that animals living in habitats with extreme seasonality may be adapted to resist change in their gastrointestinal microbiome during the active season. The compositional robustness of the microbiome may allow for time-optimal energy harvesting needs. Further, the unchanging diversity of the microbiome throughout the active season also suggests a highly diverse GI microbial functional repertoire that through transcriptomic responses, as reported elsewhere (32), may optimally address environmental/dietary seasonality. Future work involving the effect of temperature on the metatranscriptomic dynamics of the *T.c.triunguis* GI microbiome is warranted. Although there were no significant changes induced by temperature in the *T.c.triunguis* microbiome at the community level, we detected significant experiment group-driven differential abundance of a few dozen individual lineages or ASVs (Figure 5). Despite these few taxa exhibiting differential abundance driven by temperature, their net contribution to community-wide alpha diversity and ordination clustering is negligible.

At week 2 (24°C ambient temperature), there was a total of 16 and 21 differentially enriched lineages in the experimental and control group, respectively (Figure 5A). No obvious enrichment trends were observed in terms of the number or taxonomic affiliation of these lineages. Given that there was no difference in temperature between the control and experimental group at this time point, we consider this observation to reflect minor methodological or biological variance in our data.

At week 4 (28.5°C ambient temperature) the experimental group had experienced an increase of 3.1° C mean body temperature for two weeks relative to controls. Here a clear enrichment trend emerged: 75 lineages were significantly enriched in the controls relative to only 5 enriched in the experimental group (Figure 5B). This trend indicates that most differentially abundant taxa were disproportionally depleted in the experiment group (with increased temperature) and therefore enriched in the control group. This observation, despite being inconsequential to community-level diversity metrics, supports the notion of a net decrease in abundance of 75 microbiome members in response to a sustained average temperature increase of only 3.1°C.

At week 6 (33°C ambient temperature) the experimental group experienced an additional step increase in temperature of 3.7°C for two weeks (i.e.: a net increase of 6.8°C relative to controls over the previous two weeks). Here, a similar pattern as observed in week 4 was evident: more lineages (78 total) were depleted from the experimental group (enriched in the controls) while only 19 lineages were enriched by the sustained temperature increase (Figure 5C).

Within the thermal and temporal range of our experiment (two week-spaced stepwise increases in temperature by 3.4°C), many lineages were disproportionally depleted in abundance by rising temperatures. Perhaps longer experimental time windows (> 4 weeks) or more drastic temperature increases (>6.8°C) could have resulted in more dramatic differential abundance trends leading to potential impacts on community-level metrics.

Interestingly, one of the 19 temperature-enriched lineages observed at week 6 is a member of the *Erysipelothrix* spp. (Figure 6). Members of this genus are ubiquitous Gram-positive bacteria that can infect a wide variety of hosts such as mammals, reptiles, fish, birds, and even insects (33). The genus is comprised of several species, the most notable is *Erysipelothrix rhusiopathiae,* causing significant clinical disease in livestock and has zoonotic potential. Classically *E. rhusiopathiae* is the causative agent of swine erysipelas resulting in significant losses in outbreaks due to acute septicemia, endocarditis, cutaneous lesions, and chronic arthritis (34). It is also a pathogen of zoonotic concern causing erythematous cutaneous lesions (erysipeloid) and possible fatal endocarditis if left untreated (35, 36). Zoonotic infection with *E.rhusiopathiae* is most commonly as a result of handling infected animals (37). This pathogen notably has also caused clinical disease and zoonotic transmission in American alligators (*Alligator mississippiensis*) and American crocodiles (*Crocodylus acutus*) (38), and has been isolated from a common snapping turtle (*Chelydra serpentina*) (39). Phylogenetic analysis (Figure 6) of the sequences recovered here placed the week 6 experimentally enriched *Erysipelothrix* spp. *s*equence within a clade that is most similar to previously sequenced *E.rhusiopathiae*. Although considered environmentally ubiquitous, the ASV sequenced in this experiment was most abundant relative to controls in the experimental group at the highest temperature. Enrichment of this ASV at this temperature may be expected since *Erysipelothrix* spp. is optimally incubated at 37°C (36, 40). This supports the likelihood that this ASV would appear in the experimental group at the highest temperature step.

## Conclusion

To our knowledge, this study is the first to characterize the cloacal microbial community of a semi-aquatic turtle (*T.c.triunguis*). Our results suggest a level of innate robustness in response to rapid temperature changes, perhaps related to the natural life history of *Terrapene* spp. in North America. Although there were no significant community-wide changes in abundance and diversity in response to temperature increases covering a total range of 9°C over a 6-week experiment, we detected some temperature-driven differentially abundant lineages. One such lineage, with increasing abundance with temperature, was identified as a member of the *Erysipelothrix* spp. which contains many pathogenic species. This observation suggests that the increases in environmental temperature such as those induced here may contribute to enrichment of potential pathogens. Overall, our works indicates that some members of the microbiomes of ectotherms such as *T.c.triunguis* could potentially be affected by increasing environmental temperatures. Future studies to support the innate robustness of *T.c.triunguis* utilizing metabolomics, transcriptomics, proteomics, or gene expression of the microbiome are required to confirm our hypothesis.

## Data Availability

The datasets presented in this study can be found in online repositories. The names of the repository/repositories and accession number(s) can be found at: https://www.ncbi.nlm.nih.gov/genbank/, SRX19164472-SRX19164523.
