# Supplementary material for: Microbiome resilience of three-toed box turtles (Terrapene carolina triunguis) in response to rising temperatures
Source: Front Vet Sci. 2024 Sep 2;11:1276436. doi: 10.3389/fvets.2024.1276436 (PMC11402899; doi:10.3389/fvets.2024.1276436)
Supplement: Supplementary file 1 [file Data_sheet_1.docx]

Supplemental Materials for

***Microbiome resilience of three-toed box turtles (Terrapene carolina triunguis) in response to rising temperatures***

Jimmy Guan^1^, Gustavo A. Ramírez^1,2^, Curtis Eng^1^, Brian Oakley^1^

^1^ College of Veterinary Medicine, Western University of Health Sciences, Pomona, California, United States of America

^2^ Department of Biological Sciences, California State University Los Angeles, Los Angeles, California, United States of America

Corresponding Author: Brian Oakley

boakley@westernu.edu

Figure S1. Stacked bar plots of relative abundance at the phylum level for samples collected between control and experimental at each time point. Predominant phyla present between all samples are: *Firmicutes, Bacteroidetes, and Proteobacteria.*

Figure S2. Boxplot summaries of alpha-diversity (observed ASVs) for experiment groups (red and green for control and experiment, respectively) across all time points. No significant differences between the means (student t-test, alpha = 0.05) were detected between groups at any collection time.


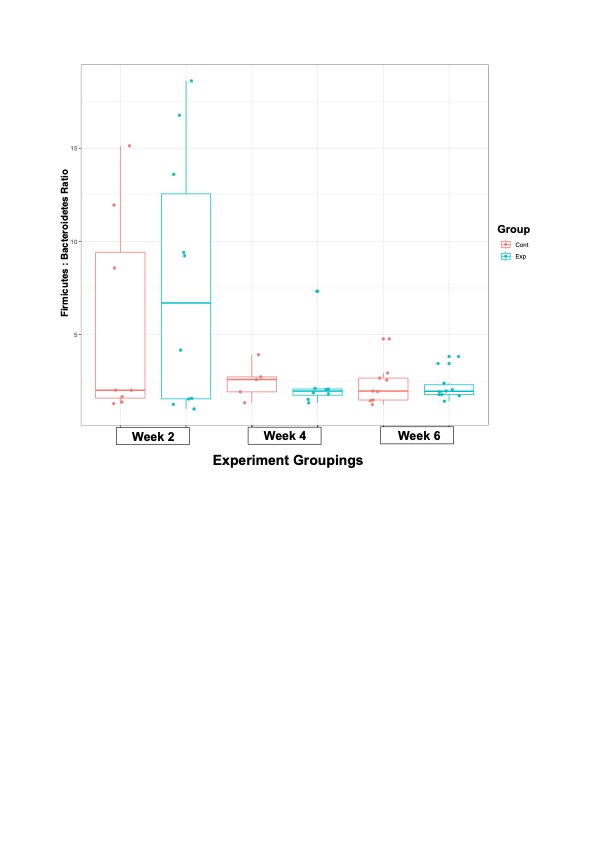


Figure S3. Boxplot summaries of F:B ratios for experiment groups (red and green for control and experiment, respectively) across all time points. No significant differences between the means (student t-test, alpha = 0.05) were detected between groups at any collection time.

| Turtle ID | Beginning Weight (g) | End Weight (g) | Mean Weight (g) | Standard Deviation |
| --- | --- | --- | --- | --- |
| 0 | 444 | 477 | 464.58 | 21.55 |
| R1 | 329 | 352 | 348.17 | 6.9 |
| R2 | 526 | 537 | 525.83 | 11.05 |
| R3 | 457 | 446 | 455.5 | 5.27 |
| R5 | 297 | 328 | 329.5 | 4.12 |
| R6 | 436 | 469 | 462.46 | 12.44 |
| R7 | 321 | 322 | 322.92 | 6.71 |
| R8 | 318 | 344 | 338.5 | 7.32 |
| R9 | 400 | 404 | 405.23 | 3.79 |
| R10 | 355 | 373 | 378.25 | 8.69 |
| R11 | 403 | 400 | 405.46 | 11.42 |
| R12 | 336 | 347 | 350.08 | 16.48 |
| L1 | 466 | 479 | 476.75 | 6.96 |
| L2 | 422 | 443 | 440.17 | 9.81 |
| L4 | 444 | 461 | 460.25 | 14.49 |
| L6 | 372 | 390 | 394.58 | 4.17 |
| L8 | 415 | 451 | 445.17 | 11.64 |
| L10 | 446 | 457 | 462.67 | 4.38 |
| L12 | 410 | 387 | 399.83 | 9.4 |

Table S1. Weights of control and experimental *T.c.triunguis* at the beginning of the experiment, end of the experiment, mean weight, and standard deviation. White boxes are control group and gray boxes are experimental group.
